# José Francisco M. Salomão — President of the ISPN 2019–2021

**DOI:** 10.1007/s00381-021-05311-8

**Published:** 2021-08-11

**Authors:** Wolfgang Wagner

**Affiliations:** Pediatric Neurosurgery, University Medical Center, Johannes Gutenberg-University, Mainz, Germany

**Fig. 1:**
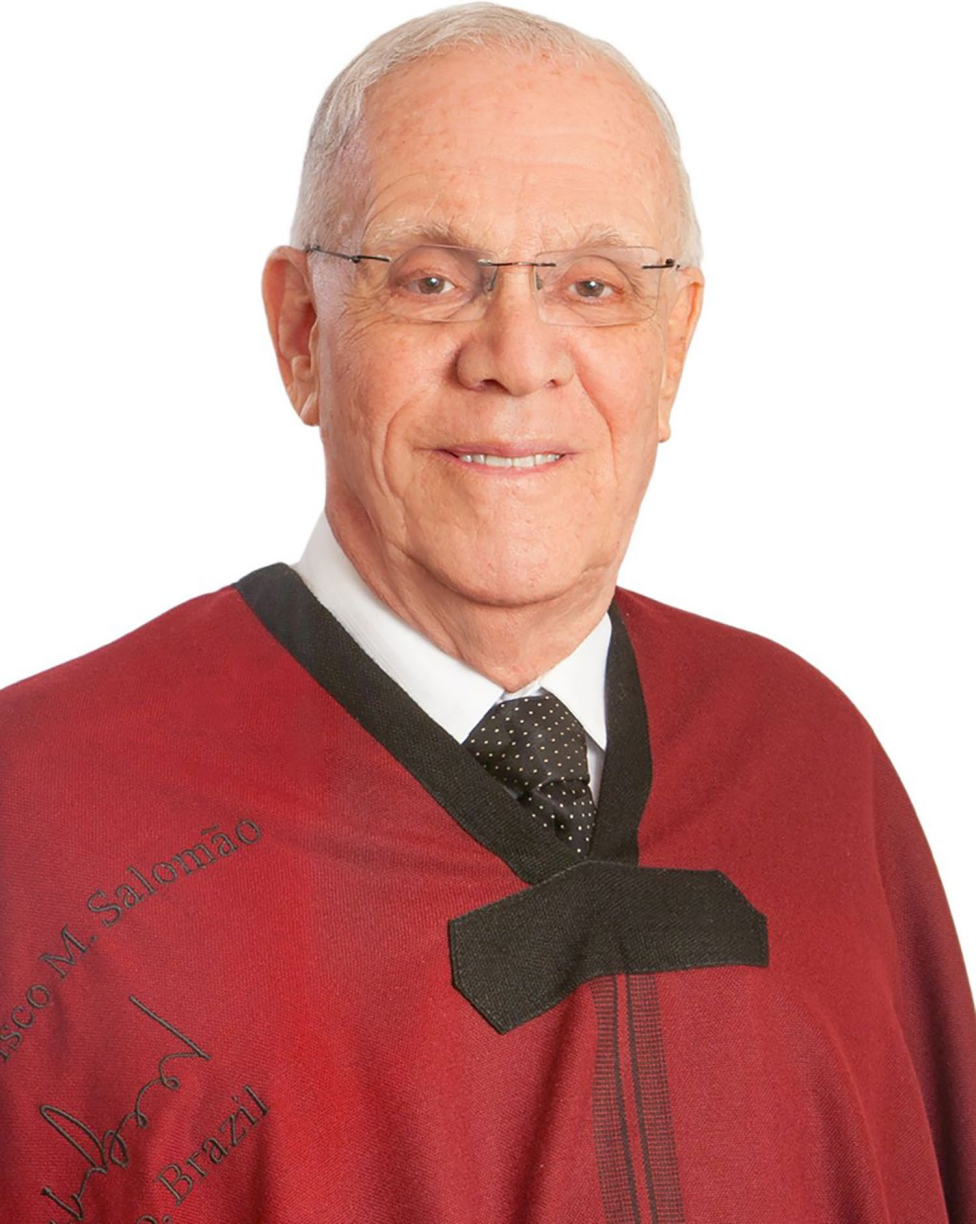
José Francisco M. Salomão, the president of the ISPN 2019–2021

José Francisco Manganelli Salomão, the president of the ISPN 2019–2021 (Fig. 1), was born on May 8, 1946, in Porto Alegre (the Portuguese name for “Joyful Harbor”), the capital of the Brazilian state of Rio Grande do Sul in the extreme south of Brazil, as the first of two brothers.

Initially, he studied there at a German school, but in 1958, the family moved to Rio de Janeiro, where his father became a general manager in the pharmaceutical industry for over 40 years. In Rio de Janeiro, Francisco completed his studies at a Franciscan College.

**Fig. 2:**
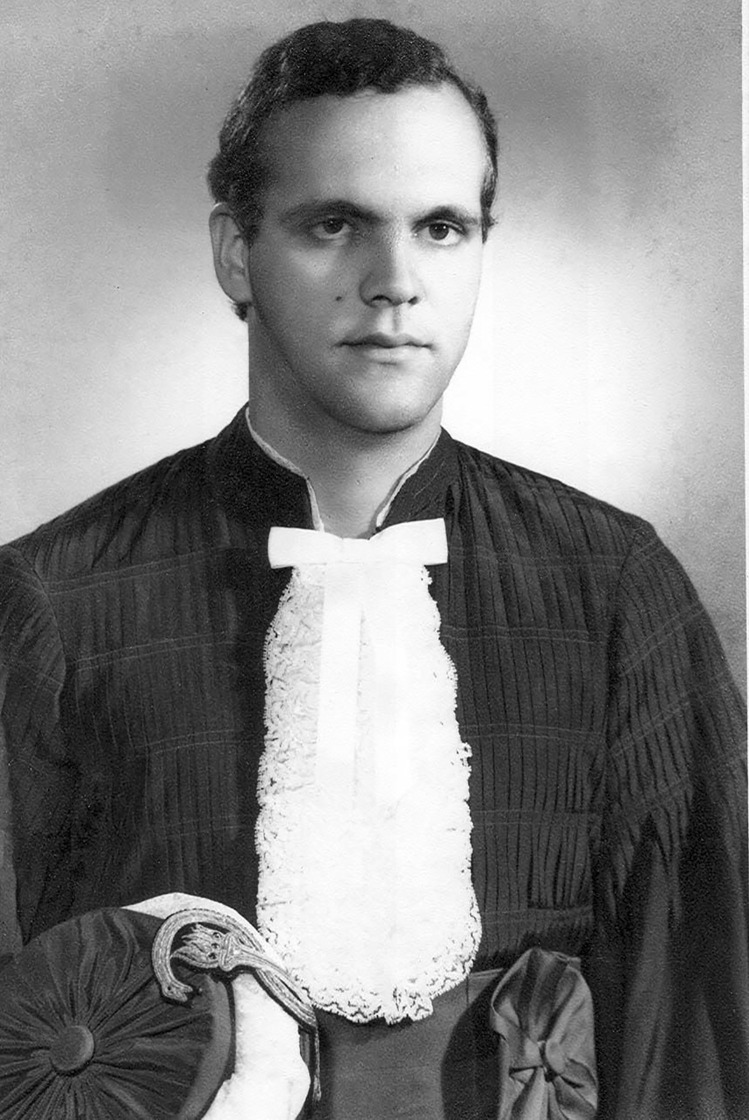
Graduation at Medical School in 1970

In 1965, he entered the University of Rio de Janeiro’s Medical School and graduated in 1970 (Fig. 2). During this time, he worked in a pharmacology lab where he became acquainted with scientific medical research — an experience that would influence his future career a lot. In the last year at the medical school, he did an internship in neurology.

In 1971, Francisco started his neurosurgical training at the Federal Hospital of the State Servers (Hospital Federal dos Servidores do Estado, HFSE) in Rio de Janeiro — a highly renowned and appreciated center of attraction and a magnet for newly graduated doctors from all over Brazil. His chief and mentor there was J. Portugal Pinto who had worked 5 years with William James Gardner at the Cleveland Clinic in Ohio. Prof. Pinto deeply influenced Francisco and aroused his interest in dysrhaphic malformations — a topic that would over the years become one of Francisco’s favorite subjects of scientific research and clinical work. In 1974, after the 3 years of medical training, he was selected for a position of Assistant Physician at the Division of Neurosurgery in the same hospital. One year later, Francisco got the position of Collaborating Professor at the Federal University in the city of Niteroi, situated across the Bay of Guanabara. There, he stayed until he was appointed to the higher position of a Chief of the Clinic at the HFSE Neurosurgery Service in 1982.

Francisco’s medical training and clinical and scientific education stayed not confined to his home country Brazil. In 1976, he spent a month at the Kantonsspital (the university hospital) in Zurich, directed by the famous Prof. Gazi Yaşargil, where he learned microsurgery techniques. In 1978, he became a Foreign Invited Assistant to the Neurosurgery Department at the University of Freiburg in Germany, where he worked mainly with Prof. Fritz Mundinger, one of the pioneers of stereotactic neurosurgery in Germany, and also with Prof. Wolfgang Seeger, well known through his illustrations of neuroanatomical structures and the topography of neurosurgical approaches. One year and a half later, Francisco returned to Rio de Janeiro. As he was still the youngest in his department, he was more or less “automatically” charged with the care of pediatric neurosurgical cases (a phenomenon well known to all of us and definitively not limited to Brazil). Nevertheless, he had also a great interest in vascular neurosurgery that made him visit Prof. Charles Drake’s service in London, Ontario in 1982.

He got the title of Master of Science in Neurosurgery and the doctor’s degree in medicine (PhD), respectively in 1996 and 2001 at the Federal University of São Paulo. This was for him a period of great academic activity, during which he initiated and supervised a considerable number of master and doctoral theses, published over 50 scientific papers in peer-reviewed journals, wrote nearly 20 book chapters and gave almost 200 oral presentations and invited lectures at congresses and scientific meetings.

As in his hospital the demand for taking care for pediatric cases increased, so did his interest in operating children. Consequently, Francisco felt the need to further increase his knowledge and to improve his clinical skills in this particular field. That became all the more important when in 1989 he was appointed chair of the Pediatric Neurosurgery Division at the Instituto Fernandes Figueira (IFF), the Maternal and Children Hospital of the Oswaldo Cruz Foundation (Fiocruz), one of the most important and traditional research institutions in Brazil. For this reason, he started visiting world-leading pediatric neurosurgery services in the Americas and Europe, including the Hôpital Necker–Enfants malades in Paris, the Hôpital de la Timone in Marseille, and the New York University Hospital in NYC.

**Fig. 3:**
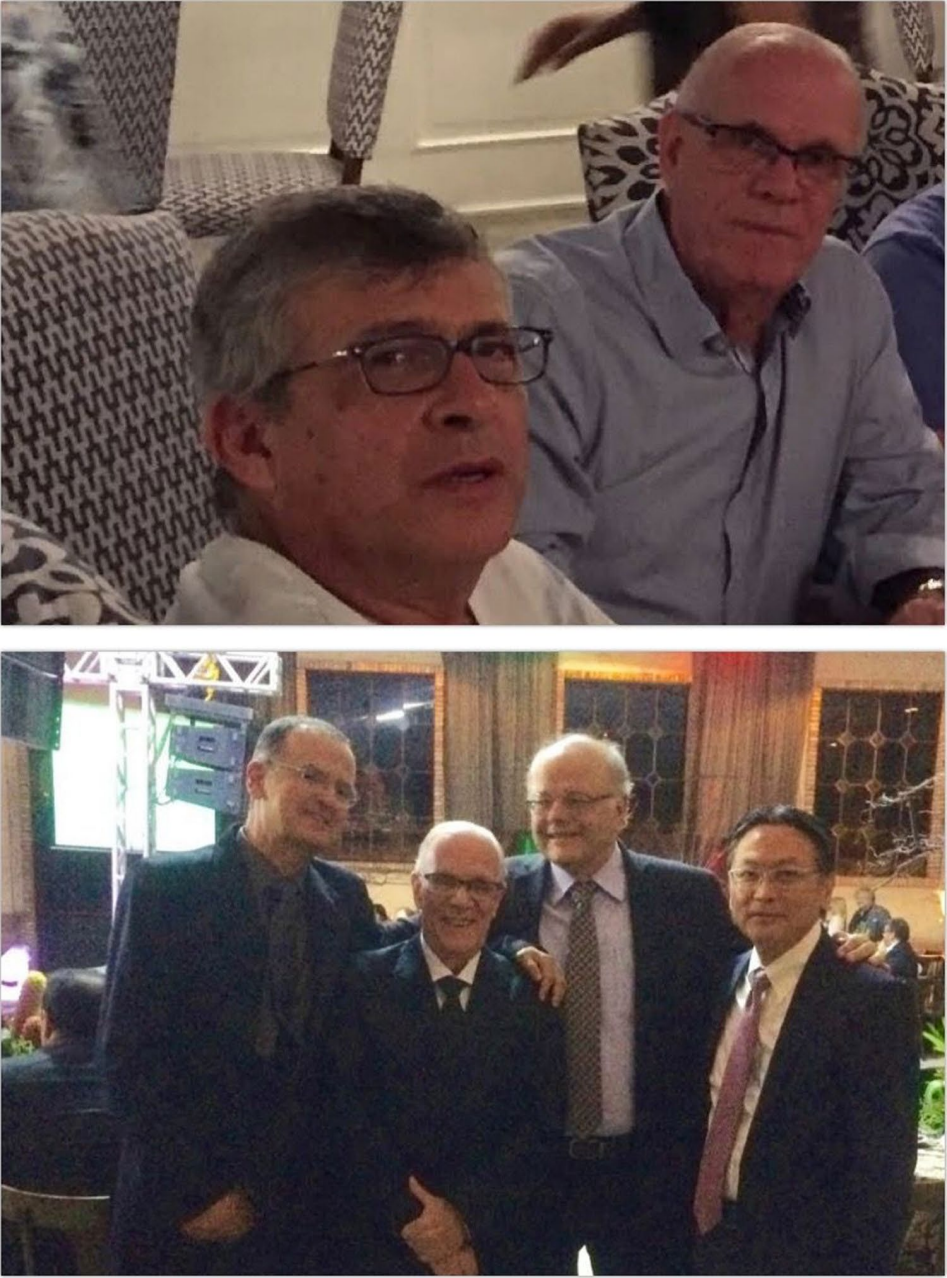
The G5: With Sergio Cavalheiro (upper) and with Benicio Lima, Helio Machado, and Hamilton Matushita (lower)

In the early 1990s in Brazil, a small group with a particular interest in pediatric neurosurgery was spontaneously created. Benicio Oton de Lima, Hamilton Matushita, Helio Rubens Machado, Sergio Cavalheiro, and José Francisco Salomão got together to form the so-called G5, a group that inside the Brazilian Society of Neurosurgery gave rise to the Section of Pediatric Neurosurgery and later to the Brazilian Society of Pediatric Neurosurgery (SBNPed) (Fig. 3). This group grew rapidly, as many others interested in Pediatric Neurosurgery joined over time and, consequently, Brazil became one of the five countries with the largest number of active members of ISPN.

The first ISPN meeting that Francisco ever attended was in 1977, in Guarujá, São Paulo, but that was only the starting point of attendance of these congresses. At the ISPN annual meeting 1995 in Santiago de Chile, Francisco realized how easy it was to get acquainted with those experts in the field whose work he admired since many years at a distance and so he became a regular attendant of these conferences.

**Fig. 4:**
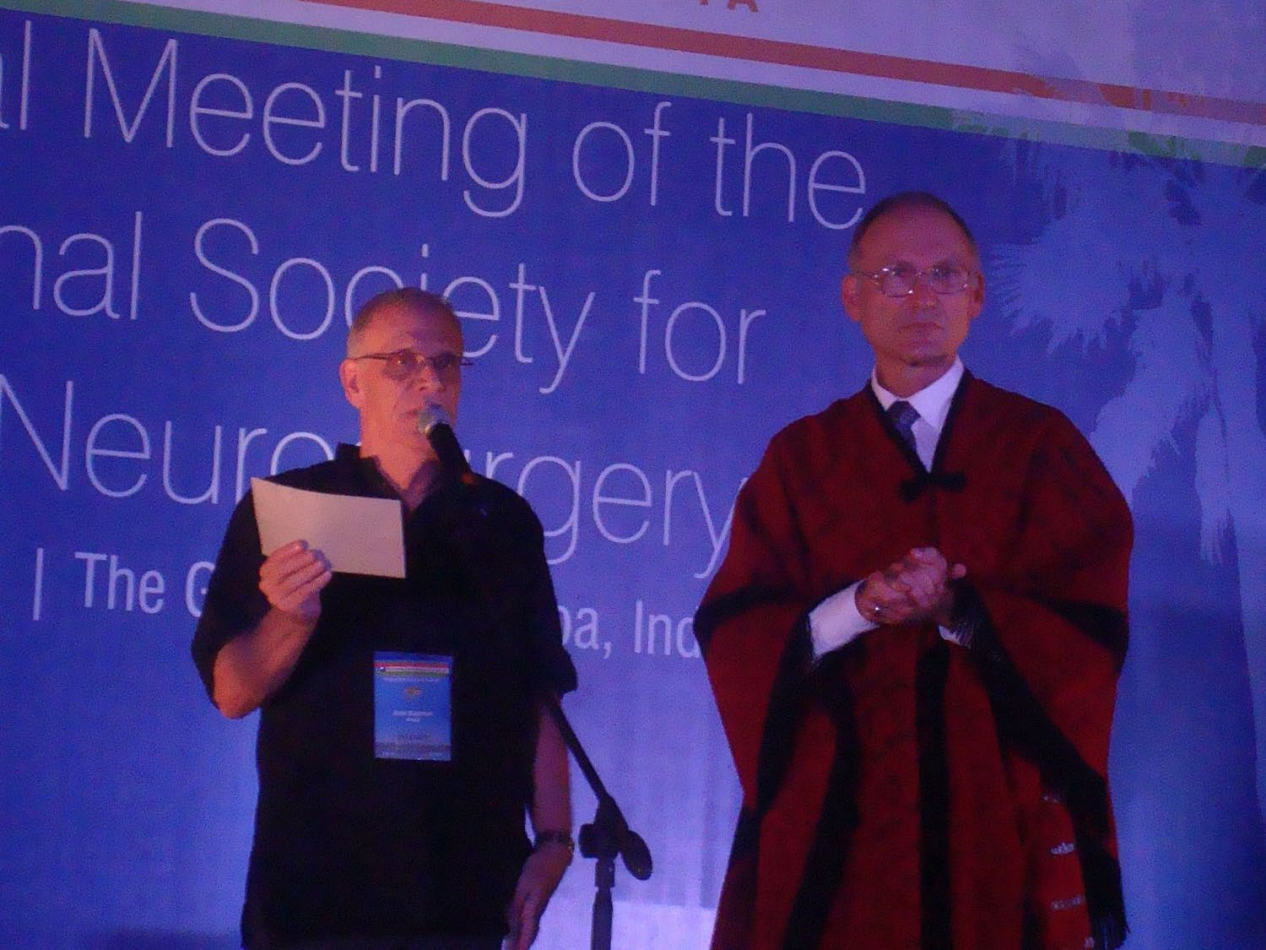
As ISPN Ways and Means Chair, with Paul Steinbok in Goa, 2011

**Fig. 5:**
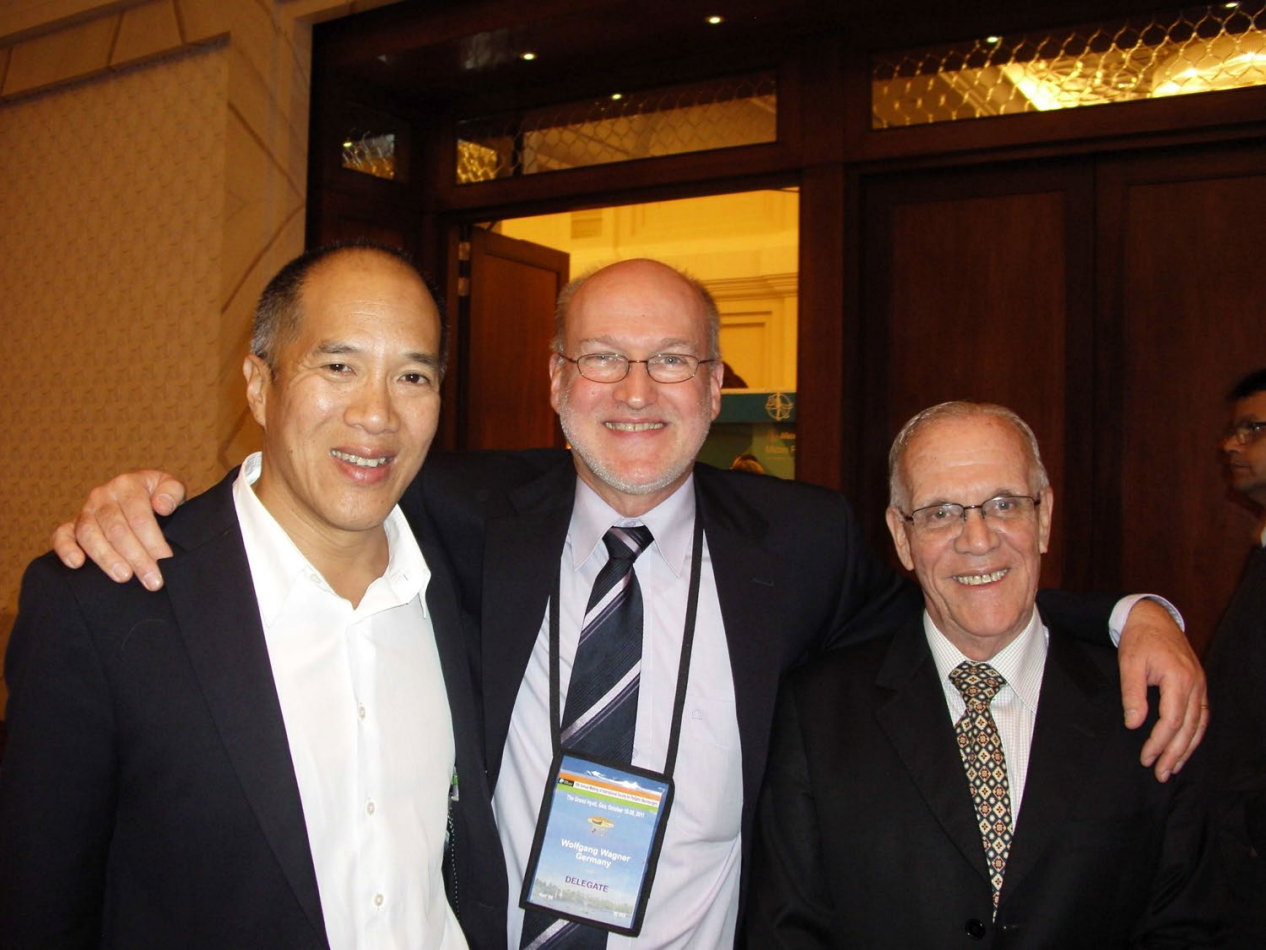
Three ISPN Annual Meeting Chairs (Sydney 2012 — Charlie Teo, Mainz 2013 — Wolfgang Wagner, Rio de Janeiro 2014 — José Francisco Salomão) at ISPN 2011 in Goa

**Fig. 6:**
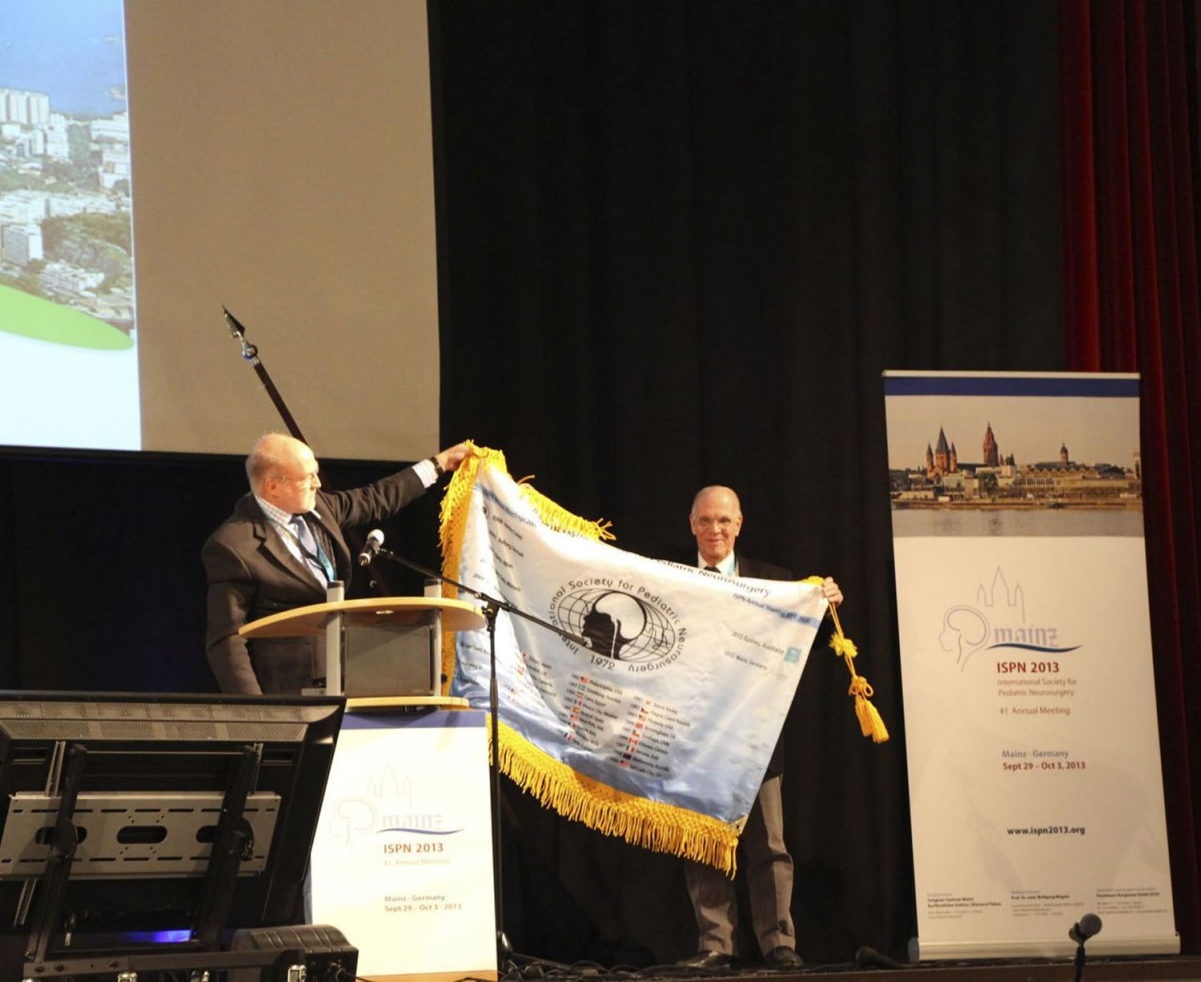
ISPN Flag Hand-Over to Rio de Janeiro in Mainz 2013

**Fig. 7:**
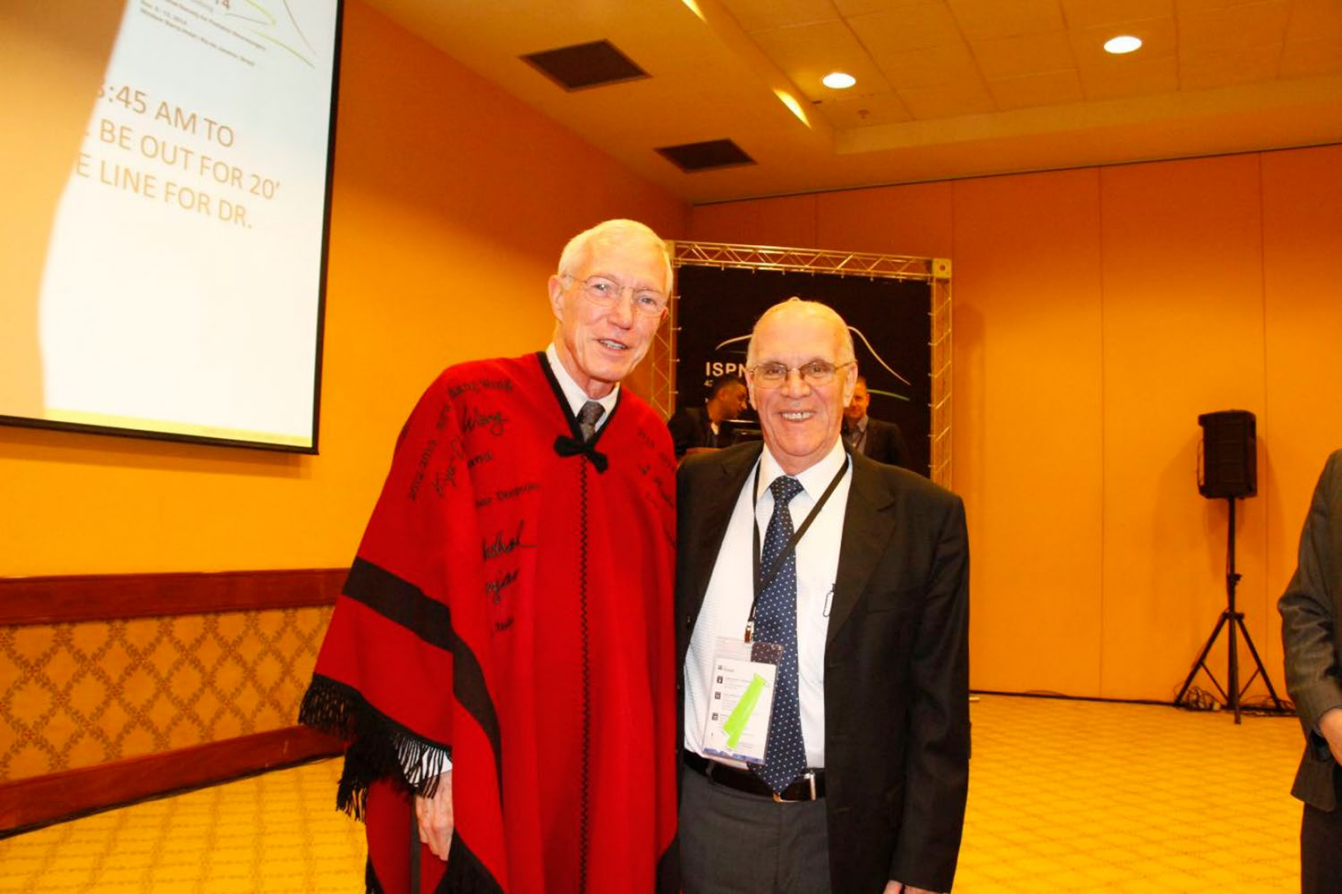
Chair of 42nd ISPN Meeting — Rio de Janeiro, with Gordon McComb, 2014

**Fig. 8:**
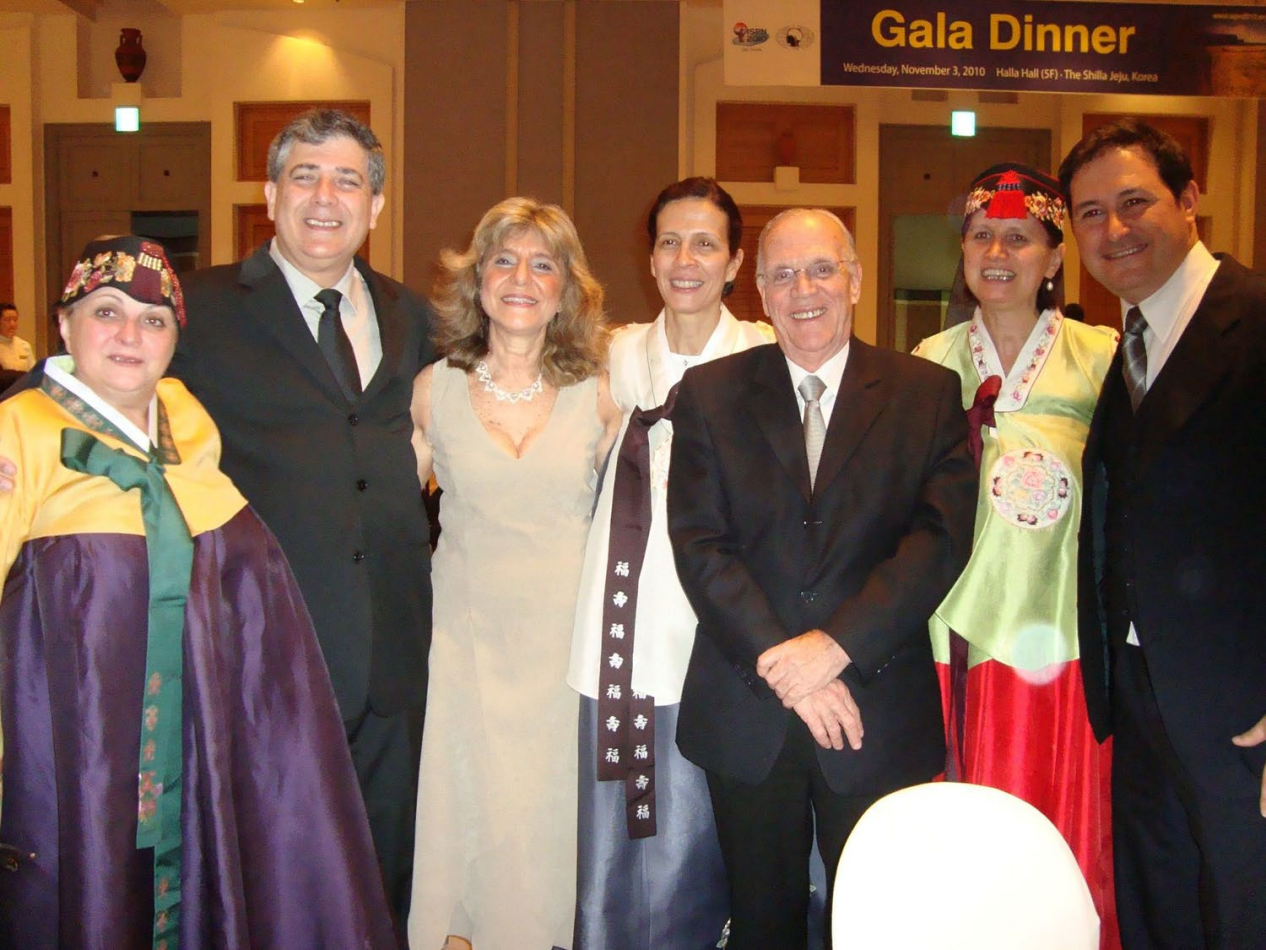
With members of the Pediatric Chapter of FLANC in Jeju, 2010. From left: Beatriz Mantese, Artur da Cunha, Graciela Zuccaro, Marcia da Silva, Nelci Zanon and Ramiro del Rio

In his professional career, Francisco successfully managed to mix academic and “networking” activities. So he held a really great number of scientific offices: President of the Neurosurgery Society of the State of Rio de Janeiro, President of the Brazilian Society of Pediatric Neurosurgery and Vice President of the Brazilian Society of Neurosurgery, to name but a few — not to forget his functions in the ISPN as chair of the Ways & Means and Audit committees (Fig. 4); in 2014, he had himself the pleasure and the honor of chairing the 42nd ISPN Meeting in Rio de Janeiro following Sydney 2012 und Mainz 2013 (Figs. 5, 6 and 7). During his presidency of the Pediatric Neurosurgery Chapter of the Federation of Latin American Neurosurgery Societies (FLANC), he deepened the friendly relationships with the Latin American leading experts in the field who formed the initial group of the current Latin American Society of Pediatric Neurosurgery, notably Graciela Zuccaro, Sergio Valenzuela, Carlos Dabdub, and Adrián Cáceres (Fig. 8). Among other projects, he created — with the support of the Brazilian Society of Neurosurgery — a supplementary Residence Program in Pediatric Neurosurgery at the Instituto Fernandes Figueira — IFF in 1990 that resulted in more than 20 pediatric neurosurgical subspecialists acting in Brazil and Latin America.

However, Francisco was always aware that medical societies have not only clinical and scientific tasks but also a social and humanitarian mission —so he can rightly be proud of having been one of those responsible for introducing the fortification of flours with folic acid in Brazil at the beginning of the twenty-first century.

**Fig. 9:**
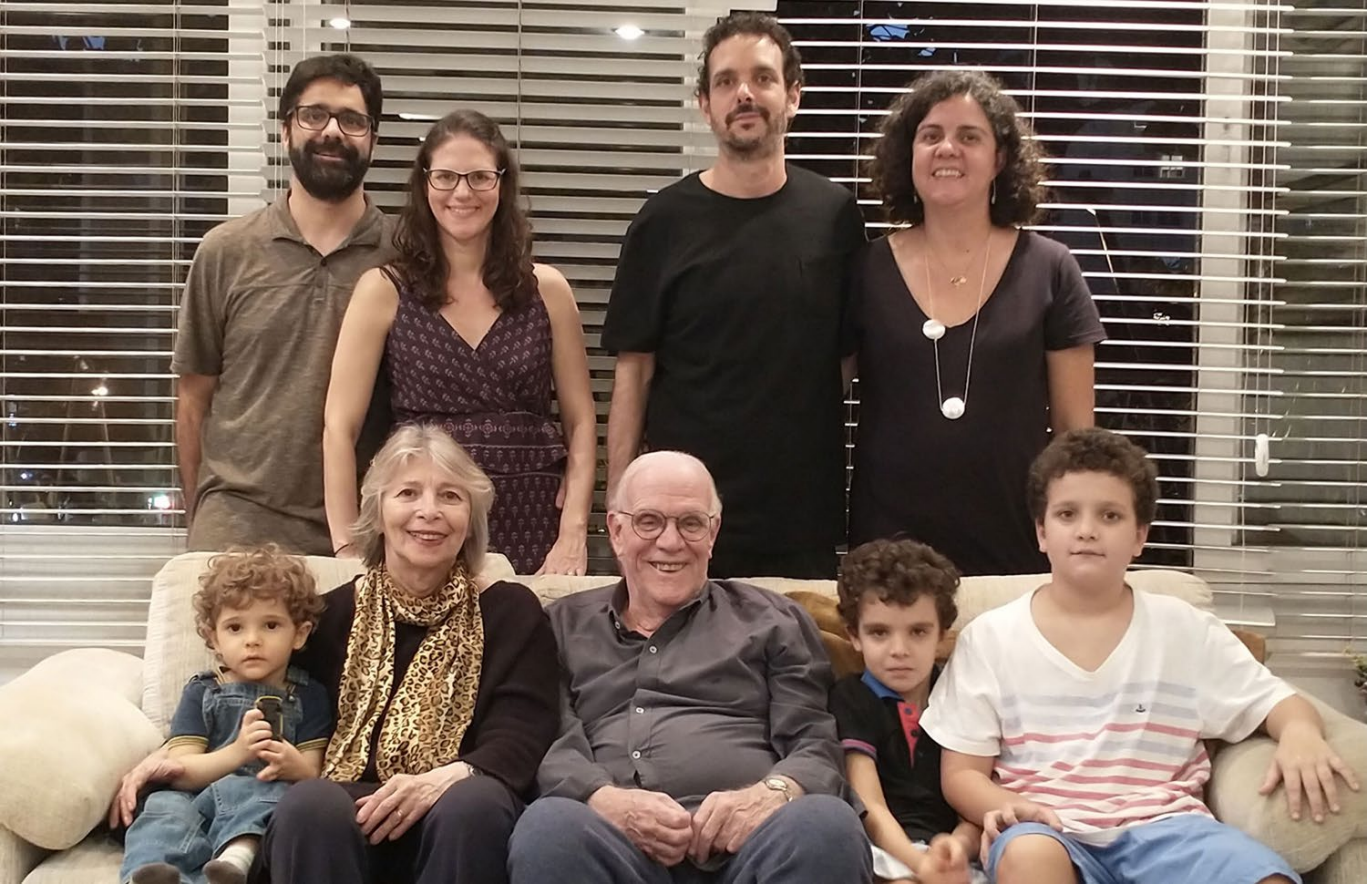
Informal family meeting after relaxation of COVID-19 restrictions, 2021

Francisco married Beatriz in 1974, not far from Rio de Janeiro, in a chapel on the farm of Beatriz’ family. They have two children, both married, and three grandchildren — all boys (not surprisingly, the Salomãos hope that at least one of them becomes interested in medicine…) (Fig. 9). Alongside his clinical and scientific activities, Francisco’s hobbies are football and music (especially jazz and — how else could you expect it from a Brazilian — bossa nova).

**Fig. 10:**
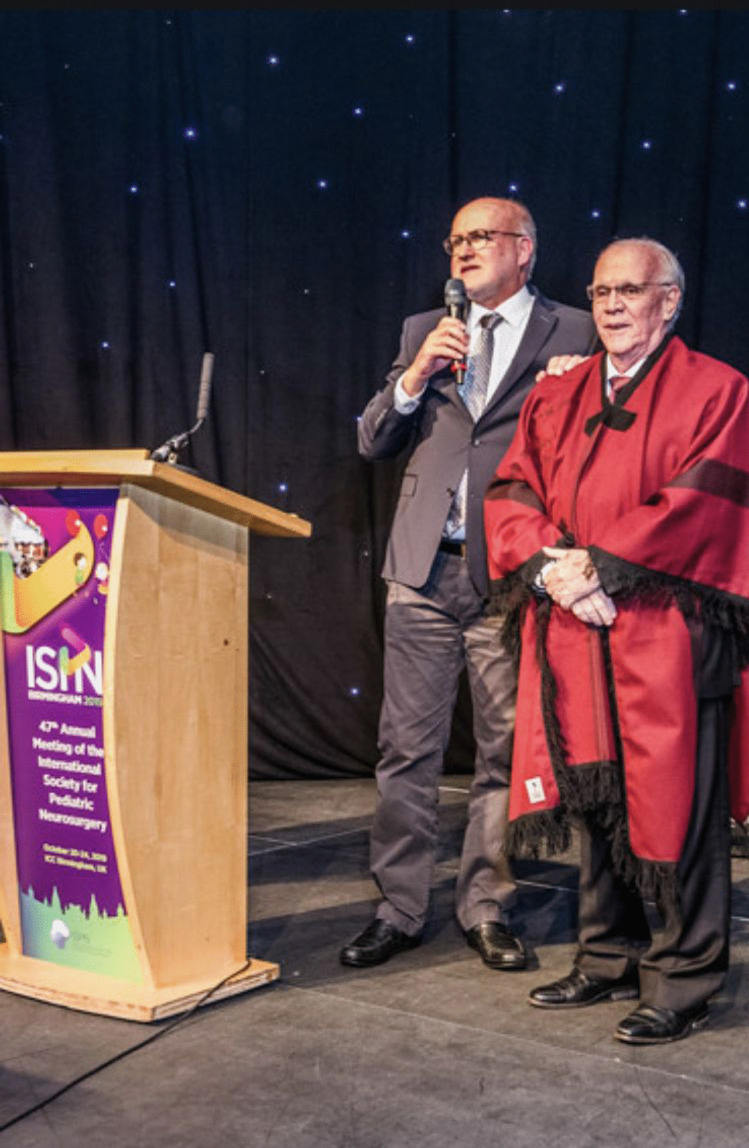
President of the ISPN in Birmingham with Wolfgang Wagner, 2019

Without a doubt the culmination of Francisco’s scientific career was his election as President-elect of the ISPN in Tel Aviv 2018; in Birmingham in 2019, he became the ISPN President (Fig. 10). His presidency fell into the time of the COVID-19 pandemic which changed so many plans not only in the ISPN. In the end, Francisco is the first president in the history of the ISPN with a term of 2 years. However, what distinguishes and characterizes his presidency and will be remembered by all of us is not a term of office of 1- or 2-year duration, but a fair leadership, straightforwardness, trustworthiness, and openness to others. Francisco is a dedicated pediatric neurosurgeon, a committed clinician, researcher, and teacher, a successful networker, a humble character — and a good friend. In other words: this is how an ISPN president should be.

